# Emergence and Evolution of Crystallization in TiO_2_ Thin Films: A Structural and Morphological Study

**DOI:** 10.3390/nano11061409

**Published:** 2021-05-26

**Authors:** Ofelia Durante, Cinzia Di Giorgio, Veronica Granata, Joshua Neilson, Rosalba Fittipaldi, Antonio Vecchione, Giovanni Carapella, Francesco Chiadini, Riccardo DeSalvo, Franco Dinelli, Vincenzo Fiumara, Vincenzo Pierro, Innocenzo M. Pinto, Maria Principe, Fabrizio Bobba

**Affiliations:** 1 Department of Physics “E.R. Caianiello”, University of Salerno, 84084 Fisciano, Italy; odurante@unisa.it (O.D.); cdigiorgio@unisa.it (C.D.G.); vgranata@unisa.it (V.G.); giocar@sa.infn.it (G.C.); 2 National Institute of Nuclear Physics (INFN), Sezione di Napoli Gruppo Collegato di Salerno, 80126 Napoli, Italy; neilson@unisannio.it (J.N.); fchiadini@unisa.it (F.C.); vincenzo.fiumara@unibas.it (V.F.); vpierro@sa.infn.it (V.P.); pinto@sa.infn.it (I.M.P.); 3 Department of Engineering, DING, University of Sannio, 82100 Benevento, Italy; riccardo.desalvo@unisannio.it (R.D.); principe@unisannio.it (M.P.); 4 National Research Council-SuPerconducting and Other INnovative Materials and Devices Institute (CNR-SPIN), University of Salerno, 84084 Fisciano, Italy; antonio.vecchione@spin.cnr.it; 5 Department of Industrial Engineering, DIIN, University of Salerno, 84084 Fisciano, Italy; 6 RicLab, Limited Liability Company, Pasadena, CA 91104, USA; 7 National Research Council-National Institute of Optics, CNR-INO, 56124 Pisa, Italy; franco.dinelli@ino.cnr.it; 8 School of Engineering, University of Basilicata, 85100 Potenza, Italy; 9 Department Electrical and Information Technology Engineering, University of Naples “Federico II”, 80138 Napoli, Italy; 10 Museo Storico della Fisica e Centro Studi e Ricerche “Enrico Fermi”, 00184 Roma, Italy

**Keywords:** thin films, TiO_2_ anatase, crystallization, surface imaging, phonon lifetime

## Abstract

Among all transition metal oxides, titanium dioxide (TiO_2_) is one of the most intensively investigated materials due to its large range of applications, both in the amorphous and crystalline forms. We have produced amorphous TiO_2_ thin films by means of room temperature ion-plasma assisted e-beam deposition, and we have heat-treated the samples to study the onset of crystallization. Herein, we have detailed the earliest stage and the evolution of crystallization, as a function of both the annealing temperature, in the range 250–1000 °C, and the TiO_2_ thickness, varying between 5 and 200 nm. We have explored the structural and morphological properties of the as grown and heat-treated samples with Atomic Force Microscopy, Scanning Electron Microscopy, X-ray Diffractometry, and Raman spectroscopy. We have observed an increasing crystallization onset temperature as the film thickness is reduced, as well as remarkable differences in the crystallization evolution, depending on the film thickness. Moreover, we have shown a strong cross-talking among the complementary techniques used displaying that also surface imaging can provide distinctive information on material crystallization. Finally, we have also explored the phonon lifetime as a function of the TiO_2_ thickness and annealing temperature, both ultimately affecting the degree of crystallinity.

## 1. Introduction

Titanium dioxide (TiO_2_) is one of the most intensively studied compounds, thanks to its unique and attractive properties, such as high refractive index, chemical stability, photocatalytic activity, and self-cleaning surface. This exciting and rich set of material properties has made TiO_2_ a valuable candidate for applications in many fields, as well as for fundamental science investigations. To date, the market demand on TiO_2_-based devices for photocatalysis [1,2,3,4], sensors [5,6], optical reflective coatings for highly innovative applications [7,8] (innovative mirrors for gravitational wave interferometers, among the others [9,10,11,12]), solar cells [13,14,15], metal insulator semiconductor industry [16], self-cleaning application [17,18,19], water purification processes [20], has been systematically growing, especially for thin films and nanostructures. In addition, a constant effort has been made in setting up reliable computational techniques, mainly based on density functional theory (DFT), to predict and describe the properties of TiO_2_, not only in its crystalline forms, but also in the amorphous phase, as well as to simulate the amorphous to crystalline phase transition [21,22,23]. Indeed, while some applications (optical fibers, displays, solar cells) require amorphous materials [24,25], some others, such as phase-change memory devices, are based on the amorphous to crystalline transition [26]. Nevertheless, the theoretical modeling of amorphous materials opens several challenges: while an ideal crystal can be described as a periodic translation of a single unit cell, the amorphous phase is characterized by a lack of periodicity, which makes it difficult to build accurate models predicting electronical, optical, and structural properties [21]. However, new theoretical approaches have been developed to describe structural similarities between amorphous and crystalline phases, with specific applications to the case of TiO_2_. These models reveal that the local structure of amorphous TiO_2_ resembles that of its crystalline counterpart: the Ti atoms prefer bonding with six O neighbors, forming an octahedral structure, and the O atoms with three Ti atoms’ neighbors [21]. Herein, we present an experimental approach to investigate the amorphous to crystalline transition of TiO_2_ thin films, by exploring the effect of post-deposition heat-treatments. This study arises in the context of a specific scientific application of amorphous coatings concerning the development of dielectric mirrors, with low transmittance and thermal noise, for the detection of gravitational waves. The in-depth knowledge of the structural and morphological evolution induced by post-deposition annealing, especially in thin films, is of fundamental importance for the development of optimized and nano-stratified coatings [10,11]. We study the onset of crystallization and its evolution, as a function of both film thickness, in the range 5–200 nm, and annealing temperature, varying between 250 and 1000 °C. Despite the use of a quite standard procedure for inducing crystallization, we have thus performed a systematic study devoted to both crystallization onset and evolution as a function of (i) material thickness and (ii) post-deposition treatments, which has established the bases for a detailed TiO_2_ phase diagram describing the amorphous-to-crystalline phase transition. The amorphous TiO_2_ thin films have been produced by room temperature electron-beam evaporation, assisted with ion-plasma bombardment to enhance material uniformity and density [27,28]. This energetic process is known to improve the material packing density, as well as the mechanical and optical properties of the films [29]. Besides electron-beam deposition [30], commonly used techniques for both amorphous and crystalline TiO_2_ thin films are RF Magnetron Sputtering [31], Chemical Vapor [32], and Pulsed Laser [33] Deposition. Among the others, we chose e-beam for its versatility and wide use in producing thin films.

The optical and electronic properties of TiO_2_ are related to its three crystalline phases, each of them characterized by a different structural order. The crystallization onset temperature of each phase is strongly dependent on the deposition method, substrates, and material form (thin films, nanoparticles, nanostructures) [34,35,36,37,38]. Commonly, the first stage of crystallization occurs in the anatase phase (A-TiO_2_), which is characterized by a tetragonal cell with: lattice parameters *a* = *b* = 0.3785 nm and *c* = 0.9514 nm; density *d* = 3.9 g/cm^3^; and optical band gap of 3.2 eV. Increasing the annealing temperature, two more crystalline phases may nucleate: brookite (B-TiO_2_) and rutile (R-TiO_2_). The B-TiO_2_ shows an orthorhombic unit cell with: lattice parameters *a* = 0.9184 nm, *b* = 0.5447 nm, and *c* = 0.5145 nm; density *d* = 4.13 g/cm^3^; and optical band gap of 3.3 eV. Finally, the R-TiO_2_ has a tetragonal unit cell with: lattice parameters *a* = *b* = 0.4594 nm and *c* = 0.2958 nm; density *d* = 4.23 g/cm^3^; and optical band gap of 3.0 eV [30]. The A- and B-TiO_2_ are considered as metastable phases that may convert into the R-phase as a consequence of thermal processes [31].

In the present paper, we report on the amorphous to A-TiO_2_ phase transition, studied by means of Atomic Force Microscopy (AFM), Scanning Electron Microscopy (SEM), X-ray Diffractometry (XRD), and Raman Spectroscopy (RS). In particular, we use AFM and SEM to study TiO_2_ surface changes, in terms of roughness, particle size, and morphological features, as a function of both film thickness and heat-treatments. We show a clear evolution of the surface features toward a crystalline order, starting around the crystallization onset temperature (*T*_c_), as measured consistently by both XRD and RS. Finally, we exploit the capabilities of RS in studying more specific aspects of the lattice dynamics, such as phonon lifetime, which is known to a have great impact on optoelectronic device performances [39]. Analyses of phonon lifetime have been thus carried out as a function of both thickness and annealing temperature.

## 2. Materials and Methods

### 2.1. Fabrication

TiO_2_ thin films, with nominal thickness of 5, 32, 64, 100, and 200 nm, were fabricated using the OAC75F coater equipped with an electron-beam evaporation source by Optotech (Wettenberg, DE). A quartz crystal monitor (QCM) was used to determine the deposition rate (typically 1 Å/s in the investigated samples) and to stop the fabrication once the desired thickness was reached. The electron gun was operated at 8 kV, while a mixture of argon and oxygen, with a flow rate of 5 sccm and 20 sccm, respectively, was used. The growing material was continuously bombarded with an ion flow (ion-plasma assist used power was 800 W), in order to enhance the material compactness (the pressure during the ion assisted deposition was 10^−4^ mbar).

Polished silicon substrates, 1” or 2” in diameter and 0.5 mm or 1 mm in thickness, were sonicated in acetone, isopropyl alcohol (30 min each), deionized (DI) water, and blow-dried with ionizing air gun, prior to loading them into the deposition chamber. The substrates were placed in a dome holder rotating at 25 rpm speed, ~70 cm distant from the source, and the chamber was evacuated down to a pressure of 10^−6^–10^−7^ mbar.

### 2.2. Characterization

#### 2.2.1. Thermal Annealing

The as deposited TiO_2_ thin films have been heat treated in air. The heater is placed in a stainless-steel chamber and is proportional-integral-derivative (PID) controlled. The thermal process takes place in three steps, i.e., (i) heating ramp with a fixed rate, (ii) plateau at the desired temperature setpoint, and (iii) cooling ramp with a fixed rate.

The samples were heated with a rate of 3 °C/min, up to a preset temperature of 250, 300, 400, 450, 500, 600, 800, 1000 °C, and left at fixed *T* for 12 h. Finally, they were cooled down to room temperature with a rate of 1 °C/min.

#### 2.2.2. Atomic Force Microscopy

AFM images have been acquired with a Nanowizard III, equipped with Vortex electronics from JPK (Berlin, DE), in the standard tapping mode technique, using a SCM-PIT-V2 tip from Bruker (Billerica, MA, USA). The cantilevers used were characterized by means of force curve spectroscopy and thermal methods, resulting in a resonance frequency and elastic constant of *f*_0_ ≈ 75 kHz and *k* ≈ 3 N/m, respectively. The AFM images were treated to remove the artifacts due to the bending of the piezo scanner. The raw data were subjected to a standard background subtraction, and tilt removal, as well as to a de-spiking procedure, when needed. The root-mean-square (RMS) roughness, defined as the root mean square of the height distribution of the sampled area, has been chosen to describe the surface morphology. All the data have been analyzed by using the *WSxM* 5.0 software by NanoTec, 2015. 

#### 2.2.3. Scanning Electron Microscopy

The surface morphology was analyzed by a field emission scanning electron microscope (FESEM) from ΣIGMA Zeiss (Jena, DE), with a nominal resolution of 1.3 nm at 20 kV. The high-resolution SEM micrographs have been acquired using an InLENS detector with an acceleration voltage of 10 kV, a working distance of 5 mm and a beam current of 80 µA. The film composition X-ray microanalysis was performed by energy dispersive spectrometry (EDS) using an Oxford-Inca Energy 300 system (Abingdon-on-Thames, UK) to check chemical composition and absence of contamination in as deposited and annealed films.

#### 2.2.4. X-ray Diffraction

In order to study the structural properties, X-ray diffraction analysis was performed on as-grown and annealed TiO_2_ thin films. All the samples have been routinely analyzed using a diffractometer from Bruker (Billerica, MA, USA). In addition, *θ*–2*θ* scans with a time/step 0.2–0.6 s, a 2*θ* step of 0.02°, and a 2*θ* angle ranging from 20 to 75° were performed to check the emergence of crystallinity as a function of annealing temperature. X-ray analysis with a high-resolution Philips X-Pert MRD diffractometer (Eindhoven, NL) was also carried out using monochromatic Cu Kα1 radiation with wavelength of 0.154056 nm. The diffractometer was equipped with a four crystal Ge220 asymmetric monochromator and a graded parabolic mirror positioned on the primary arm which reduces the incident beam divergence to 0.12 arc sec. *θ*–2*θ* scans were done with time/step of 100 s, 2*θ* step of 0.02°, and 2*θ* angle in the range from 20° to 75°.

Post deposition analyses, based on X-ray reflectivity (XRR), were performed to confirm the effective thickness of the investigated samples, together with profilometry, by using Alpha-Step D-600 (Milpitas, CA, USA). The XRR analysis performed on the 5 nm sample (reported in Figure S1 of Supplementary Materials, Section S1), showed an actual thickness of (5.14 ± 0.02) nm; similarly, the 100 nm sample resulted in being (98 ± 5) nm under profilometric measurements. These results confirmed that the actual thickness is always close to the nominal one.

#### 2.2.5. Raman Spectroscopy

A Raman Spectrometer (RENISHAW InVia, Wotton-under-Edge, UK), in the backscattering geometry, was used to exploit the structural and vibrational properties of the investigated samples. A near infrared laser, with a wavelength of 785 nm, was used as the excitation source. We acquired large range spectra (100–1200 cm^−1^) by using a 50× magnification, 10 s exposure time, 5% laser power, and 5 accumulations, and small range spectra (100–200 cm^−1^), by using a 50× magnification, 10 s exposure time, 10% laser power, and 3 accumulations. We acquired multiple (≥5) Raman measurements, and we averaged the results.

## 3. Results and Discussion

The structural and morphological properties of amorphous TiO_2_ thin films, with nominal thickness of 5, 32, 64, 100 and 200 nm, have been routinely characterized soon after the deposition (as-grown) as well as after each heat treatment, to follow the evolution of TiO_2_ crystallization as a function of thickness and annealing temperature.

### 3.1. Study of as-Grown TiO_2_ versus Thickness

**Figure 1:**
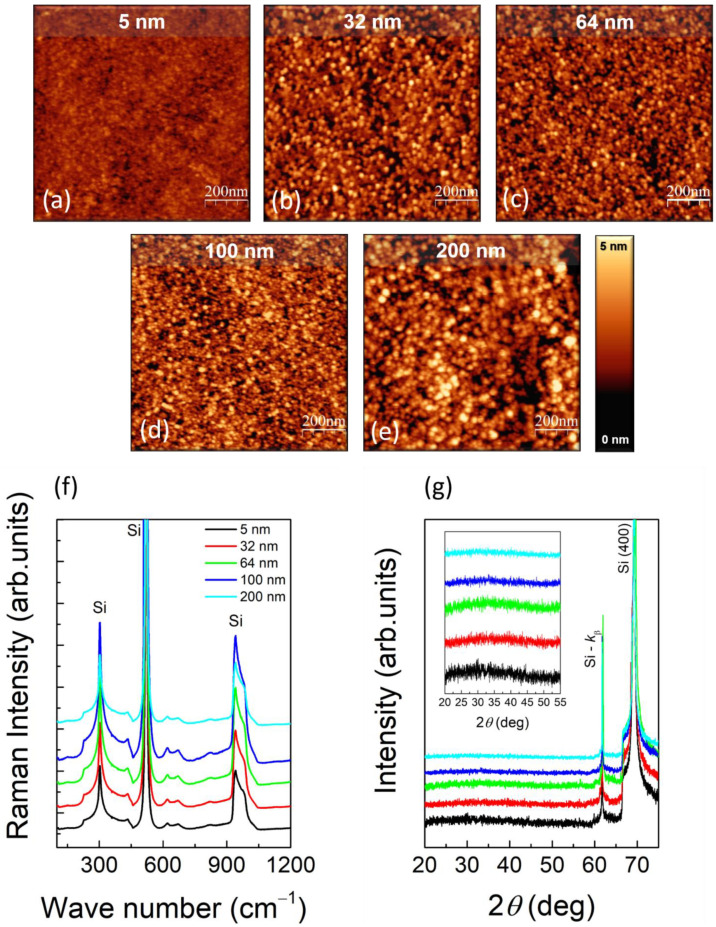
(**a**–**e**) Tapping-mode AFM topography, 1 μm × 1 μm in size, of as-grown TiO_2_ samples; (**f**) RS spectra—wavenumber range from 100 to 1200 cm^−1^; (**g**) XRD spectra—2*θ* range from 20 to 75° (main) and from 20 to 55° (inset). Black, red, green, blue, and cyan curves refer to 5, 32, 64, 100, and 200 nm. The curves have been offset along the *y*-axis for better readability.

Figure 1a–e show representative tapping-mode AFM morphologies, 1 μm × 1 μm in lateral size, of the as-grown samples as a function of the TiO_2_ thickness, from 5 (Figure 1a) up to 200 nm (Figure 1e). A black-to-white color scale has been used to highlight the surface roughness, and, to favor a direct comparison, all the images have been equalized to the same color contrast, from a minimum value of 0 (black) to a maximum of 5 nm (white).

One can easily observe that the surface of all the as-grown samples is made of particles. Those particles, more and more evident as the thickness increases, form in the amorphous phase, as demonstrated by Raman spectroscopy (RS) and X-ray Diffraction (XRD) (Figure 1f,g, respectively). In these plots, different colors have been used to refer to different TiO_2_ thickness values: black, red, green, blue, and cyan curves refer to 5, 32, 64, 100, and 200 nm, respectively. For clarity of representation, the curves have been offset along the *y*-axis. It is worth noting that all the Raman modes observed in Figure 1f are associated to Si. Besides the three highest Raman bands clearly indicated in Figure 1f, the features appearing at 460 cm^−1^ and from 618 to 675 cm^−1^, having lower intensity, are also attributed to Si [40]. Similarly, only the peaks relative to the Si substrate show up in the XRD spectra of Figure 1g (main), together with a broad bump in the 2*θ* range from 20° to 45° (as shown in the inset), representative of the amorphous TiO_2_.

**Figure 2:**
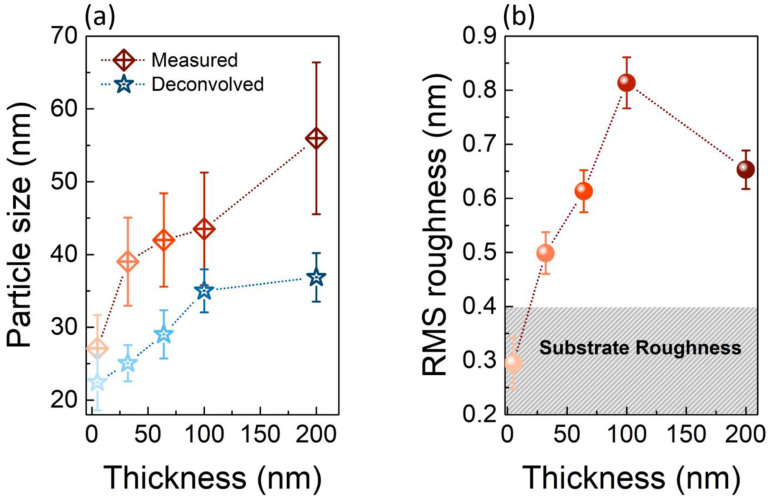
(**a**) Measured (reddish scatters) and deconvolved (bluish scatters) particle size, in the as-grown samples; (**b**) average RMS roughness as a function of the TiO_2_ thickness. Dashed lines are used both in (**a**,**b**) as a guide for the eye. In (**b**), the dashed grey area corresponds to the range of the measured Si substrate roughness.

We have then performed a statistical analysis of the AFM images to study both the particle size and the RMS roughness as a function of the TiO_2_ thickness. Figure 2a displays the plot of the particle size versus the thickness, both as measured and after a deconvolution procedure (reddish and blueish scatters, respectively). A clear monotonic trend is shown by both, with the measured particle size ranging from (27 ± 5) nm for the 5 nm sample to (52 ± 8) nm for the 200 nm one. However, in the approximation of a spherical shape, the measurement of the particle size with AFM is commonly affected by two factors: (i) convolution between particle and tip size (which causes a non-negligible size overestimate, more pronounced as they become comparable); (ii) high packing density (which, on the contrary, causes a particle size underestimate, because only the top-most cap of each particle is exposed at the surface, and thus measured with AFM).

We have taken into account both the finite size of the tip, with a nominal curvature radius of *r* = 25 nm, and the packed nature of the measured topographies, to evaluate a deconvolved value of the particle size. Using the following Equation [41]:

This is example 1 of an equation:

(1) $$r=\frac{{\left(R-h\right)}^{2}}{2h},$$

where *h* is the height of the spherical caps as measured with AFM, and we have calculated the actual particle radius *R* and its size *2R*. In doing so, we have evaluated the deconvolved size for each sample, ranging from (22 ± 4) nm for the 5 nm sample to (37 ± 3) nm for the 200 nm one. Even though a correct deconvolution of the particle size is generally hard to obtain, we observe an increasing trend as the thickness increases, both for the measured as well as the deconvolved particle size. Such a trend is consistent with the expected behavior, based on Ostwald ripening [42], concerning the increasing size of stable particles for longer thin film depositions.

Figure 2b displays the behavior of the RMS roughness, calculated on an area of 1 μm × 1 μm, as a function of the TiO_2_ thickness. We observe an almost monotonic increase of the measured RMS roughness with increasing thickness, from (0.29 ± 0.05) nm for the 5 nm thick sample to (0.65 ± 0.04) nm for the 200 nm one. A tiny decrease in the RMS roughness is measured between the 100 and 200 nm samples, even though the difference in the correspondent RMSs is extremely small (~150 pm). This analysis has allowed us to infer that the deposition technique used (i) guarantees a high coverage uniformity, even for an ultra-thin deposition (5 nm), whose RMS roughness approaches the one of the substrate (the grey region in Figure 2); (ii) gives rise to a remarkable surface flatness, as the RMS roughness remains always below 1 nm.

### 3.2. Study of TiO_2_ Crystallization Onset versus Thickness

**Figure 3:**
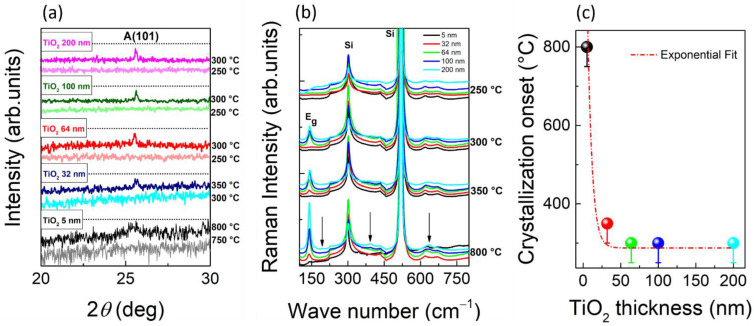
(**a**) XRD spectra of the 200 nm sample at 250 (light magenta) and 300 °C (magenta), the 100 nm sample at 250 (light green) and 300 °C (green), the 64 nm sample at 250 (light red) and 300 °C (red), the 32 nm sample at 300 (light blue) and 350 °C (blue), and the 5 nm sample at 750 (grey) and 800 °C (black). The symbol A indicates the anatase phase; (**b**) Raman Spectra of the 5 (black), 32 (red), 64 (green), 100 (cyan), and 200 (blue) samples annealed at different temperatures (250, 300, 350, and 800 °C); (**c**) crystallization onset temperature as a function of the TiO_2_ thickness. The red dashed line is the exponential fit.

In order to explore the amorphous to crystalline phase transition and study the evolution of crystallization in TiO_2_ as a function of thickness, different 12 h in-air annealing processes were carried out, with annealing plateau temperature lying in the range 250–1000 °C. The details of the heat treatments and the heating/cooling parameters are reported in the “Experimental Section 2.2.1”. Here, we want to stress that each single treatment has been performed on a pristine as-grown sample, to avoid any memory effect of multiple subsequent heating/cooling cycles. Figure 3a,b show XRD and RS measurements performed after annealing. Figure 3a shows the XRD spectra performed on each sample, right before and right after its crystallization onset temperature. In Figure 3a, a pattern of diffraction peaks related to a TiO_2_ crystalline phase is visible. In particular, we focus on the (101) reflection, at 2*θ* = 25.2°, of the anatase phase, which is expected to appear at lower annealing temperatures than brookite and rutile.

As shown in Figure 3a, we notice that the transition from amorphous to crystalline occurs between 250 and 300 °C for the 200, 100, and 64 nm samples (magenta, green, and red curves, respectively), and between 300 and 350 °C for the 32 nm sample (blue curves). Finally, a very tiny peak indicating crystallization is present in the 5 nm sample (black curve) after annealing at 800 °C. Note that, while the *y*-scale (intensity) of Figure 3a is almost the same for the 32, 64, 100, and 200 nm samples, we have zoomed the *y*-scale for the 5 nm one, for a better visualization of the peak, also visually leading to an enhanced noise level.

**Figure 4:**
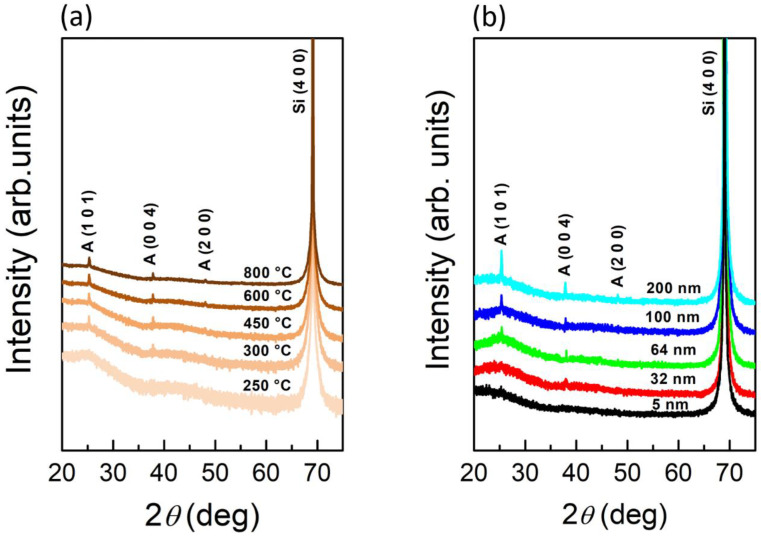
(**a**) XRD spectra of 200 nm TiO_2_ samples annealed in air for 12 h at different temperatures; (**b**) XRD spectra of TiO_2_ films with a thickness of 5, 32, 64, 100, and 200 nm annealed in air for 12 h at 800 °C. The symbol A indicates the anatase phase.

A consistent information on the crystallization onset is obtained by performing RS measurements and focusing on the appearance of the strongest anatase Raman vibrational band, the Eg mode at ~141.5 cm^−1^. We observe the appearance of the Eg anatase mode only upon annealing at 300 °C for 200, 100, and 64 nm samples (blue, cyan and green curves, respectively) and at 350 °C for the 32 nm (red curve) (Figure 3b). Less clear is the RS response of the 5 nm sample, whose behavior after annealing will be further discussed. Besides the Eg mode, additional Raman peaks show up in the RS of the 200 nm sample at the annealing temperature of 800 °C (as indicated by the black arrows in Figure 3b). The peak at 194 cm^−1^ is still attributed to the anatase Eg Raman band. The two peaks at higher wavenumbers, 394 and 637 cm^−1^, can be attributed to either anatase or brookite phases. Indeed, anatase and brookite Raman bands are expected to be very close (expected values are 399 and 639 cm^−1^ for the anatase and 396 and 636 cm^−1^ for the brookite) [37], and a non-negligible convolution effect with the Si substrate bands, located at 460 cm^−1^ and between 618 and 675 cm^−1^, can affect the exact determination of the peak centers. However, the high-resolution XRD spectrum, reported in Figure 4b, allows for excluding the presence of the brookite phase.

Finally, Figure 3c summarizes the results obtained, by plotting the crystallization onset temperature *T*_c_ versus the thickness *d*. Note that an asymmetric error bar has been used to highlight the uncertainty in the estimate of *T*_c_ due to the 50 °C step used in the heat treatments. We then fit the behavior of *T*_c_ vs. *d* by using [43]:

(2) $$T_{c}=T_{\mathrm{ac}}+\left(T_{\mathrm{melt}}-T_{\mathrm{ac}}\right)e^{-\frac{d}{\lambda }},$$

where *T*_melt_ represents the melting temperature of the bulk crystalline TiO_2_ (1843 °C), *T*_ac_ is the crystallization onset temperature of a thick amorphous film, and *λ* is the characteristic decay length of the crystallization onset temperature versus thickness. From the fit, plotted in Figure 3c as the red dashed line, we have derived *T*_ac_ = 287 °C and *λ* = 6 nm. In conclusion, we demonstrated that there is a strong dependence of the crystallization onset temperature on the sample thickness [43], i.e., it increases as the thickness decreases, especially when it is reduced to very few atomic layers, consistently with previously published results on the magnetron sputtered TiO_2_ films [44].

### 3.3. Study of TiO_2_ Crystallization Evolution versus Thickness

To get some insights into the structural changes induced by the annealing treatments, high resolution XRD spectra have been acquired for the 200 nm film as a function of the annealing temperature (in the range 250–800 °C) (Figure 4a), and as a function of the thickness at a fixed temperature of 800 °C (Figure 4b). Starting from 300 °C, the XRD peaks corresponding to the anatase reflections (101), (004) and (200) are present in the 200 nm film, as clearly indicated in Figure 4a. Performing a Debye–Scherrer analysis, and averaging the results in the temperature range considered, we find a mean TiO_2_ crystallite size of ~65 nm. No signature of a crystalline phase different from the anatase one has ever been detected, upon annealing at 800 °C, in any of the investigated samples, as shown by Figure 4b. Additional XRD measurements are reported in Figure S2a–e of Supplementary Materials, Section S2, for annealing at different temperatures as a function of TiO_2_ thickness. The occurrence of the rutile only in the thinnest samples (5 and 32 nm) and only upon annealing at 1000 °C is also discussed.

**Figure 5:**
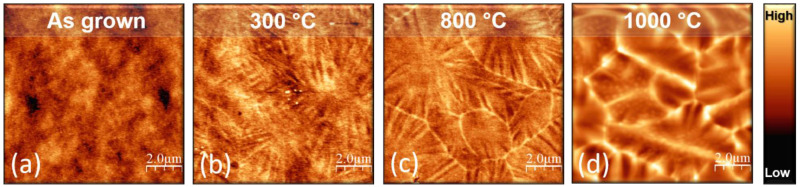
Tapping mode AFM topography, 10 μm × 10 μm in size, of TiO_2_ 64 nm (**a**) as-grown and annealed at (**b**) 300, (**c**) 800, and (**d**) 1000 °C. The color scale ranges from 0 to 6 nm in (**a**–**c**) and from 0 to 80 nm in (**d**).

Morphological changes associated with the amorphous to crystalline transition, as well as with the crystallization evolution, have been further investigated with AFM and SEM. In particular, Figure 5 reports the thermal evolution of the surface morphology in the 64 nm sample (similar analyses performed on the other samples are shown in Table S1 of Supplementary Materials, Section S3). Figure 5a–d display the tapping-mode AFM topography, 10 μm × 10 μm in lateral size, of the sample as-grown (Figure 5a) and annealed at three representative temperatures: 300 (Figure 5b), 800 (Figure 5c), and 1000 °C (Figure 5d). Increasing the annealing temperature, we observe the evolution from disordered particles (in the as-grown samples, Figure 5a) to the formation of rather large micrometer-size slabs, each of them disclosing a similar substructure characterized by radial ridges (a similar material arrangement has been shown by Ta_2_O_5_ [45]).

It is worth noting that the aforementioned features do not appear at any temperature value lower than *T*_c_ (300 °C for the 64 nm). Moreover, while, at the crystallization onset, those features are barely visible (Figure 5b), they become more and more evident, as the annealing temperature increases. Indeed, they look very well formed at 800 °C (Figure 5c), and even more defined at 1000 °C (Figure 5d), where an overall densification of the material can also be observed. We conclude that the surface morphology changes in correlation with the crystallization process, starting from 300 °C for the 64 nm film. Furthermore, in agreement with recent observations reporting a preferential 2D growth of crystalline TiO_2_ [46,47], these peculiar linear corrugations are interpreted as crystalline plates, forming grains with different crystalline orientations.

**Figure 6:**
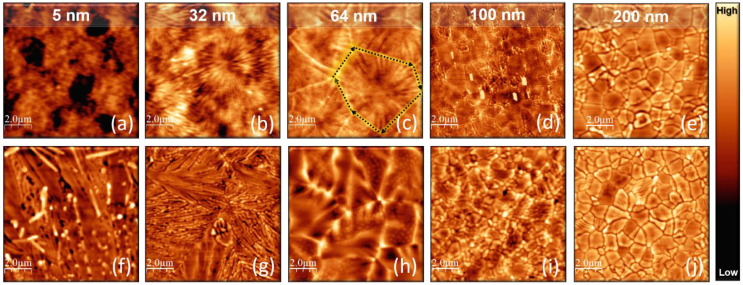
Tapping mode AFM topography, 10 μm × 10 μm in size, of: (**a**) 5, (**b**) 32, (**c**) 64, (**d**) 100, and (**e**) 200 nm samples, annealed at 800 °C. In figure (**c**), the black dotted arrows are guides for the eye highlighting grain boundaries. (**f**) 5, (**g**) 32, (**h**) 64, (**i**) 100, and (**j**) 200 nm samples, annealed at 1000 °C. The color scale of the samples annealed at 800 °C ranges from: 0 to 5 nm for 5, 64 and 100 nm; 0 to 2 nm for 32 nm; 0 to 15 nm for 200 nm. The color scale of the samples annealed at 1000 °C ranges from: 0 to 100 nm for 5 nm; 0 to 60 nm for 32, 64 and 100 nm; 0 to 80 nm for 200 nm.

However, the evolution of the surface morphology is quite different for different TiO_2_ thicknesses. Figure 6 compares representative tapping mode AFM topographies, 10 μm × 10 μm in lateral size, of all the samples annealed at the highest temperatures (800 and 1000 °C). These images show peculiar morphologies that are strongly dependent on the TiO_2_ thickness.

The annealing at 800 °C induces randomly distributed holes in the 5 nm sample (Figure 6a). The depth of these holes is (2.9 ± 0.7) nm, indicating the occurrence of a TiO_2_ partial de-wetting. On the other hand, the surface of both 32 and 64 nm samples is characterized by the appearance of linear morphological structures (see Figure 6b,c, respectively). These features seem to have a higher density in the 32 nm sample, whereas they are accompanied by the formation of more pronounced boundaries in the 64 nm (as indicated by the black dotted arrows in Figure 6c). Further increasing the thickness, the surface appears entirely cracked in plates or blocks, as shown in Figure 6d,e, as confirmed also at 1000 °C (Figure 6i,j, respectively). On the other hand, at 1000 °C, the 32 and 64 nm samples are still characterized by linear structures, even though both surfaces look morphologically denser (Figure 6g,h, respectively). Finally, the thinnest 5 nm sample presents a clear “1D-like” material reorganization upon annealing at 1000 °C (Figure 6f), resembling the local formation of nanorods (this is consistent with the observation of TiO_2_ crystallization in nanowires, at the terrace edges of a SrTiO_3_ perovskite surface, as observed in Ref. [48]). We stress that such crystalline features are visible on both the nano and macro-scale, the latter shown in the optical microscopy images reported in Figure S3 of Supplementary Materials, Section S4.

**Figure 7:**
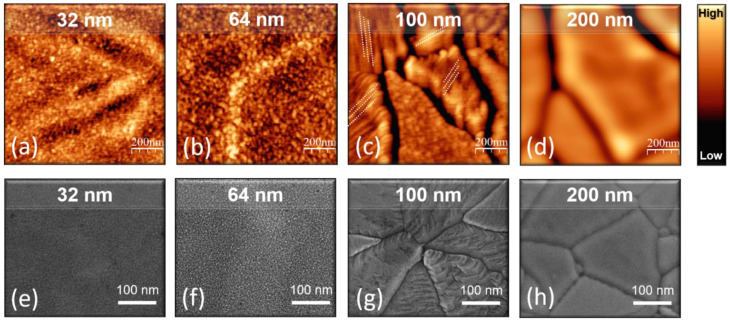
Tapping mode AFM topography, 1 μm × 1 μm in size, of (**a**) 32, (**b**) 64, (**c**) 100, and (**d**) 200 nm samples annealed at 800 °C. The color scale ranges from: 0 to 5 nm for the 32 and 64 nm samples; 0 to 20 nm for the 100, and the 200 nm samples. SEM images of (**e**) 32, (**f**) 64, (**g**) 100, and (**h**) 200 nm samples, annealed at 800 °C.

In light of this, Figure 7 details the AFM and SEM morphology of the 32 (Figure 7a,e), 64 (Figure 7b,f), 100 (Figure 7c,g), and 200 nm (Figure 7d,h) samples, on a smaller scan area, after annealing at 800 °C. From AFM, one can notice that, on a scan area of 1 μm × 1 μm in lateral size, both the 32 and 64 nm samples (Figure 7a,b respectively) still exhibit a surface made of particles, even though they seem to align along preferential directions, as evolving toward a more ordered material organization. Differently, blocks separated by deep cracks are clearly evident in the 100 and 200 nm samples. It is worth noticing that the topography of the 100 nm sample (Figure 7c) resembles unequivocally the formation of crystalline blocks, each of them with different orientation (as indicated by the white dashed lines), pointing toward: (i) the formation of a polycrystal as a consequence of the heating treatment; (ii) the tendency of TiO_2_ to crystalize preferentially into plates having the same orientation inside each block. The blocks imaged in the 200 nm sample (Figure 7d,h) are: (i) slightly larger than those imaged in the 100 nm one (Figure 7c,g), barely reflecting the trend in crystal size obtained from the Debye–Scherrer analysis of high resolution XRD spectra reported in Figure S2f of Supplementary Materials, Section S2; (ii) with a much flatter topmost average RMS of 0.72 nm, indicating a higher degree of order with increasing the thickness, in good agreement with the XRD patterns of Figure 4. SEM investigations have further confirmed the topographic features observed with AFM. Indeed, the SEM micrographs of the 32 and 64 nm samples (Figure 7e,f, respectively) show a surface made of uniformly distributed particles and a similar trend towards grain boundaries formation is also observed. On the other hand, the SEM pictures of the 100 (Figure 7g) and 200 nm (Figure 7h) samples show the development of well-defined grains with evident “plate like” crystal morphology.

Combining the AFM and SEM observations made on a large and small scan area (Figure 6 and Figure 7, respectively), we conclude that the thinnest films preserve a morphology made by individual particles, even after annealing at 800 °C, while thicker films are more prone to form crystalline grains with a more ordered organization.

It worth mentioning that we have also explored the behavior of the Si substrate, in the annealing temperature range considered, to exclude any possible influence of Si surface changes on the investigated thin films. As shown in Figure S4 of Supplementary Materials, Section S5, we find that the Si surface is almost unchanged after the heat treatments, and no stress is induced or released by the annealing (no change in the Raman vibrational mode wavenumber).

**Figure 8:**
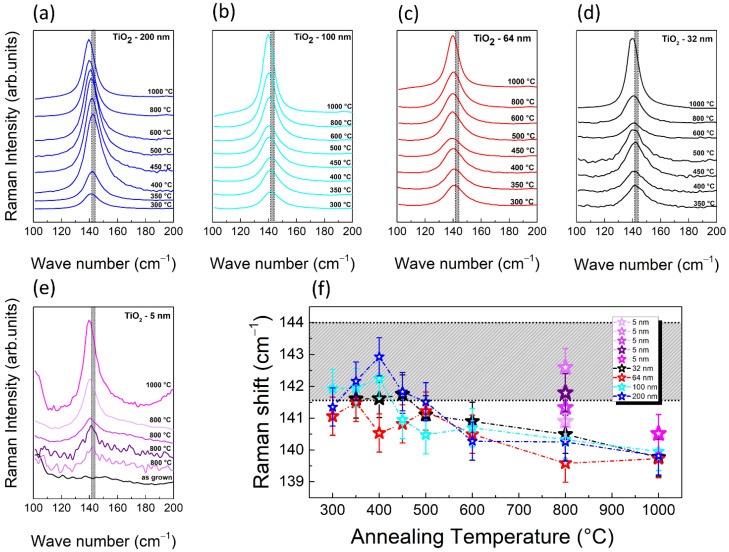
Raman spectra in the range 100–200 cm^−1^ of (**a**) 200; (**b**) 100; (**c**) 64; and (**d**) 32 nm samples at different annealing temperatures; (**e**) 5 nm sample, as-grown, annealed at 800 °C in different locations, and annealed at 1000 °C; (**f**) plot of the Raman shift as a function of the annealing temperature. The grey region highlights the range where the Eg Raman mode is expected.

Finally, we have used RS to explore the evolution of the vibrational properties of TiO_2_ as a consequence of crystallization and during crystallization evolution. In particular, we have studied both shift and linewidth of the Eg Raman mode of the anatase phase, as a function of thickness and annealing temperature. Figure 8a–e reports spectra in the region of interest (100–200 cm^−1^) as acquired for the 200, 100, 64, 32, and 5 nm samples, respectively.

Figure 8a–d show spectra acquired on the 200, 100, 64, and 32 nm samples, respectively, at different annealing temperatures, and offset along the *y*-axis for clarity of visualization. Figure 8e shows spectra acquired on the 5 nm sample as-grown, annealed at 800 °C as a function of the location, and annealed at 1000 °C. Note that enhanced laser power has been used for the spectra of the 5 nm sample annealed at 800 °C, compared to ones of Figure 8a–d, and to the one at 1000 °C. Indeed, differently from the thicker samples, the 5 nm one shows an inhomogeneous Raman response at the onset of crystallization (800 °C), with the intensity of the Eg mode being strongly dependent on the position over the sample surface. It is important to underline that, in the configuration used, RS has a higher spatial resolution compared to XRD (~1–100 μm^2^ in RS versus mm^2^ in XRD) and is thus more capable of revealing any crystallization inhomogeneity. Figure 8e compares the RS of the as-grown 5 nm sample, to those acquired in four different areas after annealing at 800 °C. A development of the crystalline Eg band can be clearly seen, compared to the amorphous case, even though the signal intensity (and, as a consequence, the signal-to-noise ratio) is strongly location dependent, pointing toward the presence of areas with different degrees of crystallization. This behavior can be explained with the partial de-wetting of the film observed with AFM (Figure 6a), after the heat treatment at 800 °C, leading to a non-uniform coverage across the sample surface. On the contrary, a uniform Raman response is found when probing the sample upon annealing at 1000 °C, as shown by the correspondent representative spectrum of Figure 8e, evidencing a complete crystallization of the whole film.

Furthermore, in order to study the vibrational properties of TiO_2_, in terms of band shift and linewidth, as a function of thickness and annealing temperature, we have treated the raw data as described in Supplementary Materials, Section S6 (Figure S5). Figure 8f shows the Eg peak position as a function of annealing temperature for the different TiO_2_ thickness values: violet, black, red, cyan, and blue stars indicate the Raman shift of 5, 32, 64, 100, and 200 nm samples, respectively. A grey dashed region is drawn to show the full range of wavenumbers, reported in the literature for the Eg anatase Raman band (independently from the laser wavelength) [37,49]. The bottom value of this region, at 141.5 cm^−1^, coincides with the expected position of TiO_2_-Eg when using an excitation source having a wavelength of 785 nm (as in our experiments). In the annealing temperature range of 300–500 °C, all the samples exhibit a wavenumber position of the Eg band scattered around the expected value (141.5 cm^−1^), whereas a trend with a slight red shift is measured when the annealing temperature is 600 °C or higher, perhaps indicating tensile strain. Indeed, the development of tensile stress has been observed upon crystallization, with a rapid increase for a reduced film thickness [50,51]. In this scenario, grain boundaries and cracks, as those imaged with AFM and SEM, can form to release the strain. However, the study of tensile stress as a function of annealing temperature and film thickness deserves further investigation, and it is out of the scope of the present paper. Again, the 5 nm sample exhibits a more complex and less homogeneous behavior in Raman shift, at the onset of crystallization. The Eg values, upon annealing at 800 °C, span over a wider range, from 140.5 to 142.5 cm^−1^, further proving that crystallization is location dependent.

**Figure 9:**
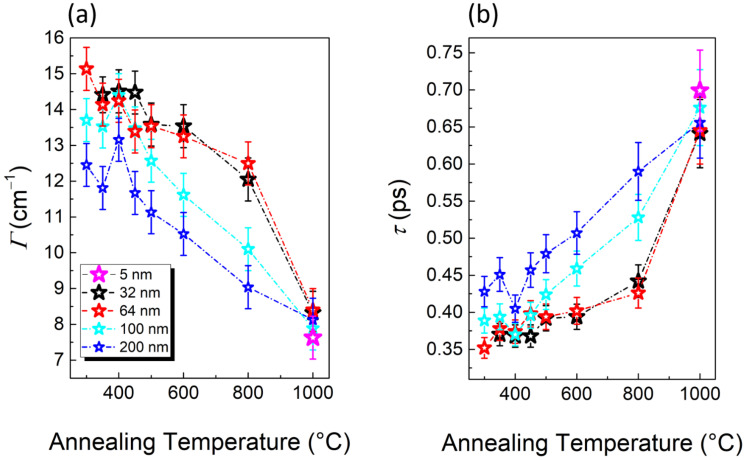
(**a**) Linewidth *Γ* values and (**b**) phonon lifetime *τ* as a function of the annealing temperature. The blue, cyan, red, and black stars indicate the 200, 100, 64, and 32 nm samples, respectively.

Finally, Figure 9a shows the plot of the Eg linewidth *Γ*, which is given by the Full Width at Half Maximum (FWHM) of the Lorentzian fit corrected by the instrumental broadening, as a function of the TiO_2_ thickness and annealing temperature. In general, the theory of a spectral line shape of crystal lattice vibrations (or phonons), in a dispersive medium, predicts the line shape to be Lorentzian and its linewidth, a parameter describing the damping effect, to be inversely proportional to the lifetime of the phonons. While in the case of an ideal harmonic crystal, the line shape is expected to be infinitesimally narrow, the experimental evidence shows that the Raman linewidth always has a finite width, indicative of the presence of decay channels, which shorten the phonon lifetimes [52,53,54,55]. Phonon shortening lifetime mechanisms are mainly attributed to: (i) crystal impurities and/or defects; (ii) anharmonic decay of a phonon into other Brillouin zone phonons. In this scenario, the phonon lifetime τ is such that:

(3) $$\frac{1}{\tau }=\frac{1}{\tau_{I}}+\frac{1}{\tau_{A}}$$

where *τ*_I_ and *τ*_A_ are the characteristic decay times due to phonon scattering at impurity sites and crystal anharmonicity, respectively. The separation of the two contributions is generally nontrivial, and can be obtained, for example, with variable temperature RS [56]. The total phonon lifetime τ is related to the Raman linewidth *Γ* as follows:

(4) $$\frac{1}{\tau }=\frac{\Delta E}{\hbar }=2\pi c\Gamma$$

where Δ*E* is the uncertainty in the energy of the phonon mode, *ħ* is the Planck constant, *c* is the light speed, and *Γ* is calculated from the FWHM and corrected by the instrumental bandpass broadening.

The spectral broadening of our spectrometer has been obtained measuring the Raman mode of Si single crystal at 520 cm^−1^, fitted by a Lorentzian line shape having an FWHM = 3.46 cm^−1^. Following Serrano et al. [57], the intrinsic TiO_2_-Eg linewidth, corrected by the instrumental broadening, has been thus estimated as:

(5) $$\Gamma =FWHM_{\mathrm{TiO}2}-\frac{FWHM_{\mathrm{Si}}{}^{2}}{FWHM_{\mathrm{TiO}2}}$$

Using this relation, we find that the instrumental broadening correction amounts to less than 7% of the measured FWHM.

Calculated *Γ* values and phonon lifetimes *τ* for each sample, at different annealing temperatures, are plotted in Figure 9a,b, respectively. As given by Equation (4), for all the samples, *Γ* decreases and *τ* increases for increasing annealing temperature. The almost monotonic increase of the phonon lifetime with the annealing temperature can be related to the evolution of crystallization, which gradually densifies the material and reduces the number of scattering centers. EDX analysis performed, for example, on TiO_2_-200 nm sample demonstrates that the material is free from external contaminations (Figure S6 of Supplementary Materials, Section S7), so that the only source of scattering can be attributed to the presence of boundaries between amorphous and crystalline areas, and between crystalline regions with different orientation and/or phase. Such an assumption is further justified by noticing that, at any temperature up to 800 °C, the phonon lifetime of the thicker samples (200 and 100 nm) is longer than the thinner ones. Indeed, the latter are still made by individual particles, even at 800 °C, and their boundaries act as scattering centers. Only, upon annealing at 1000 °C, do the phonon lifetimes measured for all the TiO_2_ thickness values studied become equal within the error bars. Interestingly, the phonon lifetime of the 200 nm sample keeps increasing smoothly within the whole annealing temperature range, while stiffer jumps are measured above 800 °C as the thickness is reduced. In particular, the phonon lifetimes of the 32 and 64 nm samples evolve almost identically, with both samples being characterized by a transition from a morphology still made by particles, at 800 °C, to a much higher densification degree at 1000 °C.

## 4. Conclusions

In the present paper, we have reported an investigation of the amorphous to crystalline transition of TiO_2_ films, varying the thickness between 5 and 200 nm. We have found that, at equal annealing conditions, the onset of crystallization occurs at a temperature *T*_c_ that becomes higher as the film is thinner. Indeed, the thickest samples (200, 100, and 64 nm) crystallize between 250 and 300 °C, whereas only a tiny feature of crystallization is found in the thinnest one (5 nm) at 800 °C. As reported in the literature, all the samples exhibit the anatase crystalline phase at the onset of crystallization. At temperatures higher than *T*_c_, we characterized the change in surface morphology with AFM and SEM, and in the Raman shift and linewidth as a function of the crystallization degree. We found that remarkable differences emerge upon varying the sample thickness. Indeed, while all the as-grown samples have a surface characterized by amorphous particles, the evolution of these topographical features, in temperature, is very peculiar of each thickness. By increasing the annealing temperature, we have noticed the progressive formation of crystalline grains, separated by deep cracks, of a few hundreds of nm in lateral size, both in the 200 and 100 nm samples. As the thickness is reduced to 64 and 32 nm, micrometer size domains with structures characterized by radial ridges appear. Finally, a partial de-wetting of TiO_2_ can be observed, together with a non-uniform crystallization, in the thinnest sample at 800 °C, followed by a whisker-like material reorganization at 1000 °C. In addition, we have investigated the behavior of the lattice dynamics, studying the evolution of the Raman Eg vibrational mode upon varying annealing temperature and thickness. While all the samples exhibit almost the same behavior of Raman shift versus temperature, with only a small red-shift at the highest annealing *T*, the value of the phonon lifetime is peculiar for each thickness, up to 800 °C. In particular, we correlated the trend of *τ*, and its higher value in the thickest films, to the densification of the material, with annealing, and a consequent reduction of phonon scattering sites. Finally, all the samples revealed the same phonon lifetime upon annealing at 1000 °C.

## Data Availability

The data presented in this study are available on request from the corresponding author.

## Supplementary Materials

The following are available online at https://www.mdpi.com/article/10.3390/nano11061409/s1, Figure S1: X-ray reflectivity (XRR) curve of an as-grown TiO_2_-5 nm film (white scatters), fitted by the blue line. Inset: cartoon representing the TiO_2_ sample on the Silicon substrate. Figure S2: XRD spectra of (a) 5, (b) 32, (c) 64, (d) 100, and (e) 200 nm TiO_2_ samples, annealed at different temperatures. Symbols A and R refer to the anatase and rutile phase, respectively. Inset of Figure S2a: zoom on the A(101) peak – annealing at 800°C. (f) Crystallite size of TiO_2_ 32, 64, 100 and 200 nm films after annealing at 800°C. The dashed line is used as a visual guide. Figure S3: Optical microscope images of TiO_2_ samples annealed at 1000 °C: (a,b) 5; (c,d) 32; (e,f) 64; (g,h) 100 and (i,l) 200 nm. Figure S4: Tapping-mode AFM topography, 10 μm × 10 μm in size, of Silicon substrate (a) pristine and (b) annealed at 1000 °C. (c) Raman spectroscopy of the Silicon substrate unheated (black) and annealed at different temperatures, in the range 250–350 cm^−1^. Figure S5: (a) Raw Raman data for TiO_2_-200 nm film annealed at 800 °C (black curve) and correspondent Silicon substrate (red curve), in the range of interest (100–200 cm^−1^). (b) Raman data of a TiO_2_-200 nm film annealed at 800 °C (black scatters) after substrate subtraction and background normalization, and the correspondent Lorentz fit (red curve). Figure S6: EDX analysis of a TiO_2_-200 nm film. Table S1: Tapping-mode AFM topography, 10 µm × 10 µm in lateral size of: 5 nm sample as-grown, and annealed at 300, 600, 800, and 1000 °C (first row); 32 nm sample as-grown, and annealed at 300, 600, 800, and 1000 °C (second row); 100 nm sample as-grown, and annealed at 300, 600, 800, and 1000 °C (third row); 200 nm sample as-grown, and annealed at 300, 600, 800, and 1000 °C (fourth row).
