# Supplementary material for: Emergence and Evolution of Crystallization in TiO2 Thin Films: A Structural and Morphological Study
Source: Nanomaterials (Basel). 2021 May 26;11(6):1409. doi: 10.3390/nano11061409 (PMC8227354; doi:10.3390/nano11061409)
Supplement: Supplementary file 1 [file nanomaterials-11-01409-s001.zip › nanomaterials-1210030-supplementary.pdf]

nanomaterials

Supplementary Materials

Emergence and Evolution of Crystallization in TiO_2 Thin Films: A Structural and Morphological Study

Ofelia Durante [1,2], Cinzia Di Giorgio [1,2], Veronica Granata [1,2], Joshua Neilson [2,3], Rosalba Fittipaldi [2,4,*], Antonio Vecchione [4], Giovanni Carapella [1,2,4], Francesco Chiadini [2,5], Riccardo DeSalvo [3,6], Franco Dinelli [7], Vincenzo Fiumara [2,8], Vincenzo Pierro [2,3], Innocenzo M. Pinto [2,3,9,10], Maria Principe [3] and Fabrizio Bobba [1,2,4,*]

1 Department of Physics "E.R. Caianiello", University of Salerno, 84084 Fisciano, Italy; odurante@unisa.it (O.D.); cdigiorgio@unisa.it (C.D.G.); vgranata@unisa.it (V.G.); giocar@sa.infn.it (G.C.)
2 National Institute of Nuclear Physics (INFN), Sezione di Napoli Gruppo Collegato di Salerno, 80126 Napoli, Italy; neilson@unisannio.it (J.N.); fchiadini@unisa.it (F.C.); vincenzo.fiumara@unibas.it (V.F.); vpierro@sa.infn.it (V.P.); pinto@sa.infn.it (I.M.P.)
3 Department of Engineering, DING, University of Sannio, 82100 Benevento, Italy; riccardo.desalvo@unisannio.it (R.D.); principe@unisannio.it (M.P.)
4 National Research Council- SuPerconducting and other INnovative materials and devices institute (CNR-SPIN), University of Salerno, 84084 Fisciano, Italy; antonio.vecchione@spin.cnr.it
5 Department of Industrial Engineering, DIIN, University of Salerno, 84084 Fisciano, Italy
6 RicLab, Limited Liability Company, Pasadena, CA 91104, USA
7 National Research Council- National Institute of Optics, CNR-INO, 56124 Pisa, Italy; franco.dinelli@ino.cnr.it
8 School of Engineering, University of Basilicata, 85100 Potenza, Italy
9 Department Electrical and Information Technology Engineering, University of Naples "Federico II", 80138 Napoli, Italy
10 Museo Storico della Fisica e Centro Studi e Ricerche "Enrico Fermi", 00184 Roma, Italy
* Correspondence: rosalba.fittipaldi@spin.cnr.it (R.F.); fbobba@unisa.it (F.B.)

.

1. X-Ray Diffraction on TiO_2-5 nm Sample

In order to confirm thickness calibration and to check the sample interface roughness, X-ray reflectivity (XRR) measurements have been performed. The XRR reflectivity acquired on a 5 nm sample is reported, as an example, in Figure S1: time step of 60 s, θ step of 0.01° and θ range from 0.15 to 3° were used. The black scatters and the blue line indicate the experimental data and the simulated curve, respectively. As a result of the fit, we obtained a total film thickness of (5.14 ± 0.02) nm and a density of (3.50 ± 0.03) g/cm^3.

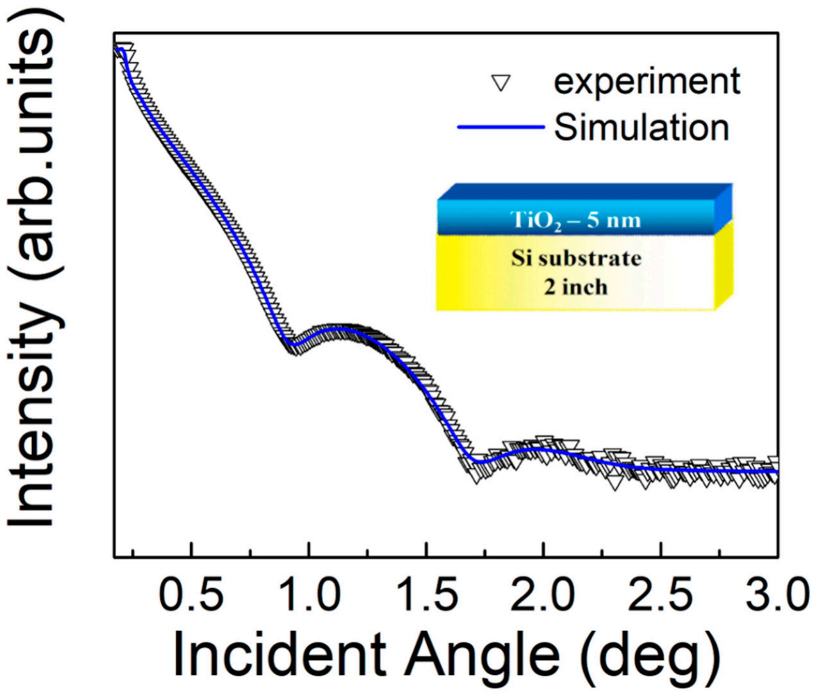

Figure S1. X-ray reflectivity (XRR) curve of an as-grown TiO_2-5 nm film (white scatters), fitted by the blue line. Inset: cartoon representing the TiO_2 sample on the Silicon substrate.

2. X-Ray Diffraction of TiO_2 Thin Films

X-ray diffraction (XRD) spectra of all the investigated TiO_2 samples, annealed at different characteristic temperatures, are reported in Figure S2a–e. The θ–2θ scans have been acquired with a time-step of 0.6 s and a 2θ step of 0.02°. All curves have been offset along the y-axis.

The XRD patterns show different peaks depending on the sample thickness. In fact, while all the films annealed at the lowest crystallization temperatures show the (1 0 1) peak of the anatase phase, only the thinnest samples (5 and 32 nm) exhibit the coexistence of the anatase and rutile phases, upon annealing at the highest T (1000 °C). Indeed, only the green XRD spectra of Figure S2a,b shows the appearance of the R(110) peak, which is instead absent in the thicker samples. It has been demonstrated that the anatase-to-rutile phase transformation depends on several factors, such as film growth technique, annealing atmosphere, level of impurities, doping, grain, and particle size [1-12]. By fixing the annealing atmosphere and the fabrication method, our samples are certainly affected by changes in grain and/or particle size as a function of thickness (Figure 2 of the main text), as well as by possible different degree of impurities and doping produced during the annealing. Indeed, the annealing is known to break the bonds between Ti and O and cause the release of oxygen from the material with the consequent formation of oxygen vacancies, as well as to possibly induce the formation of interstitial Ti atoms, especially in oxygen rich environment. Both oxygen vacancies and interstitial Ti atoms work as impurities of the crystal lattice, as well as like n-type dopants, and are thus expected to affect the anatase-to-rutile transformation [1]. The effect of crystal lattice impurities may become more and more relevant as the TiO_2 material volume is reduced, thus favoring an earlier anatase-to-rutile transition in the thinnest films (5 and 32 nm).

Additionally, the evolution of the crystallite size for films annealed at 800 °C has been investigated. In detail, the crystallite size has been evaluated from the XRD spectra shown in Figure 4a of the main text, using the Debye–Scherrer formula [13]:

$$\boldsymbol{D} = \frac{\boldsymbol{k\,\lambda}}{\boldsymbol{\beta\,cos\,\theta}} \tag{1}$$

where, D is the crystallite size, β is the full width at half maximum height (FWHM), θ is the Bragg diffraction angle, λ is the wavelength of the X-ray radiation (Cu Kα = 0.154056 nm), and K = 0.9 is the Scherrer constant (shape factor).

In our case, a Gauss peak approximation has been assumed, with $\beta = 2\sqrt{2ln2}\sigma$, σ being the gaussian standard deviation. The Scherrer analysis has been performed on the strongest anatase peak, corresponding to the (1 0 1) reflection. Figure S2f shows the trend of the crystallite size as a function of the TiO_2 thickness, upon annealing at 800 °C. An increase of the crystallite size is observed upon increasing the film thickness.

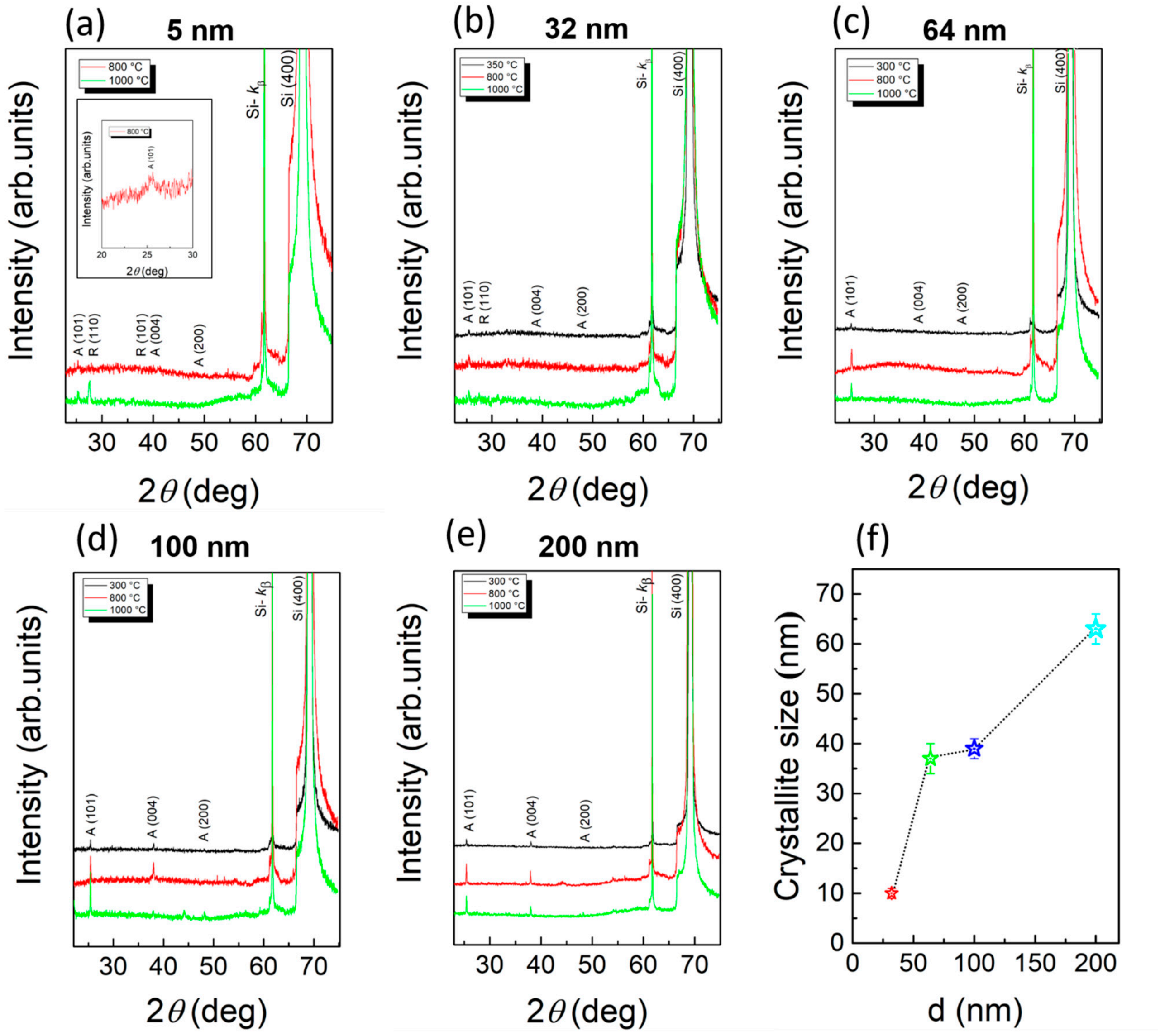

Figure S2. XRD spectra of (**a**) 5, (**b**) 32, (**c**) 64, (**d**) 100, and (**e**) 200 nm TiO_2 samples, annealed at different temperatures. Symbols A and R refer to the anatase and rutile phase, respectively. Inset of Figure S2a: zoom on the A(101) peak – annealing at 800 °C. (**f**) Crystallite size of TiO_2 32, 64, 100 and 200 nm films after annealing at 800 °C. The dashed line is used as a visual guide.

3. AFM: Temperature Evolution of TiO_2 Samples

Table S1 shows the thermal evolution of the surface morphology for the 5, 32, 100, and 200 nm thick samples. In particular, the tapping-mode AFM topographies, 10 μm × 10 μm in lateral size, are shown, for each sample after annealing temperatures at 300, 600, 800 and 1000 °C. For all the samples, a black-to-white color scale has been used to highlight the surface features.

As shown in the first row, the 5 nm sample surface looks almost unchanged after annealing at 300 and 600 °C. On the contrary, randomly distributed holes, whose depth is (2.9 ± 0.7) nm, appear at 800 °C and nanorod-like reorganization appears up at 1000 °C, as discussed in the main text.

Differently, the 32 nm sample (second row) shows a tiny modification of the surface, after annealing at 300 °C, slightly below its $T_c = 350$°C. We observe a preliminary formation of linear structures that become more and more pronounced as the annealing temperature increases, looking well formed at 800 and 1000°C.

The 100 nm sample surface (third row) evolves from a topography characterized by particle agglomerations to the formation of blocks separated by deep cracks. Those features are barely visible at 600 °C but become completely formed at 800 and 1000 °C. Their origin is discussed in the main text.

Finally, the 200 nm sample surface (fourth row) evolves from clusters of amorphous particles (as-grown) to blocks, whose size is similar to the ones measured in the 100 nm sample.

Table S1. Tapping-mode AFM topography, 10 µm x 10 µm in lateral size of: 5 nm sample as-grown, and annealed at 300, 600, 800 and 1000 °C (first row); 32 nm sample as-grown, and annealed at 300, 600, 800 and 1000 °C (second row); 100 nm sample as-grown, and annealed at 300, 600, 800 and 1000 °C (third row); 200 nm sample as-grown, and annealed at 300, 600, 800 and 1000 °C (fourth row).

**Sample**	**As grown**	**300 °C**	**600 °C**	**800 °C**	**1000 °C**
5 nm	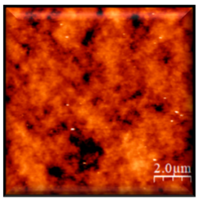	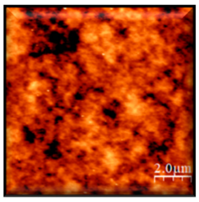	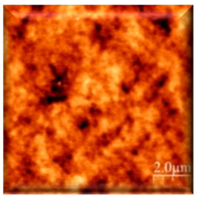	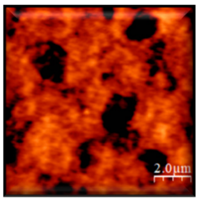	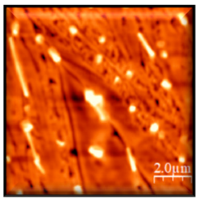
32 nm	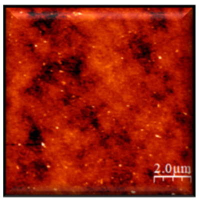	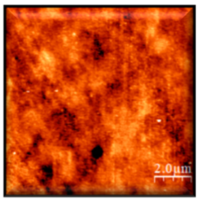	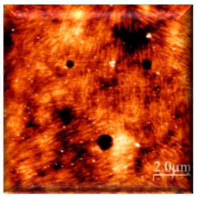	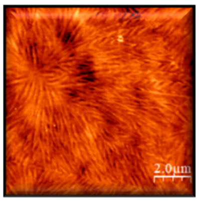	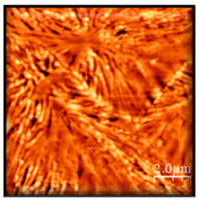
100 nm	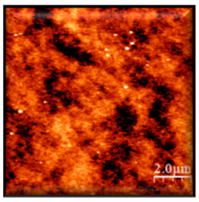	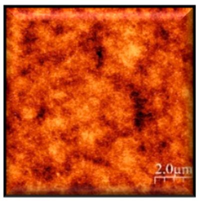	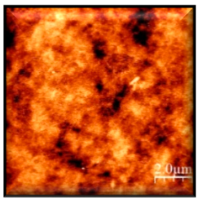	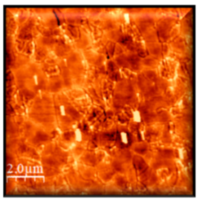	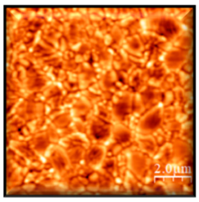
200 nm	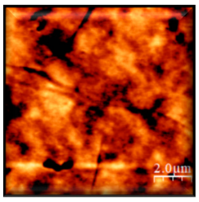	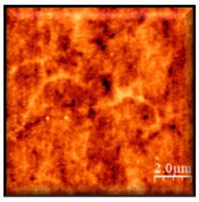	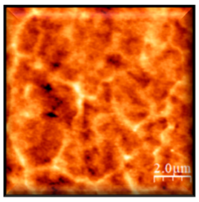	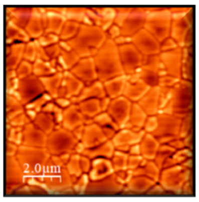	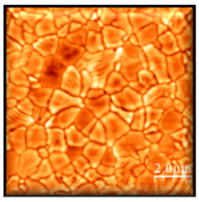

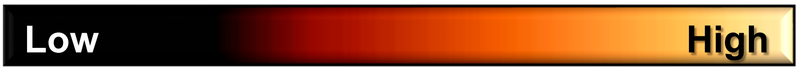

4. Optical Microscopy

The optical images display the tendency toward a "linear" material organization, after crystallization, especially evident by using a 50X magnification (Figure S3b,d,f, and g). At a lower magnification (Figure S3a,c,e,g), such linear structures give rise to a flower-like morphology, which becomes more and more compact as the thickness increases. A 5X magnification of the 100 nm sample (Figure S3g) shows the formation of profound cracks, as a consequence of material sintering. Finally, the optical images of the 200 nm samples (Figure S3i,l) show the existence of very small, separated plates or blocks, resembling those imaged by AFM and SEM.

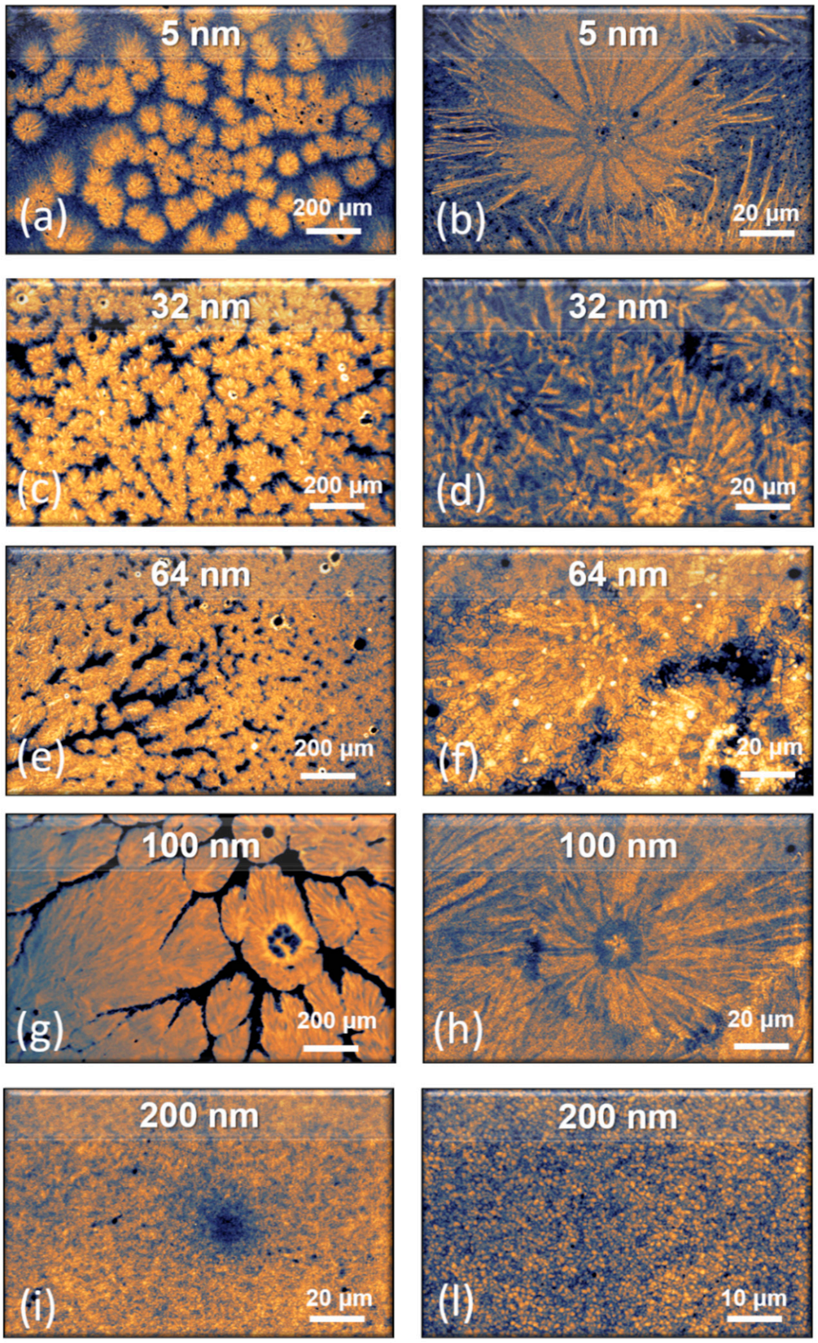

Figure S3. Optical microscope images of TiO_2 samples annealed at 1000 °C: (**a**,**b**) 5; (**c**,**d**) 32; (**e**,**f**) 64; (**g**,**h**) 100 and (**i**,**l**) 200 nm.

5. Silicon Substrate

Figure S4a,b display the tapping-mode AFM topography, 10 µm × 10 µm in lateral size, of the Silicon substrate pristine and annealed at 1000 °C, respectively. The surface appears very flat before and after the annealing process, with an RMS roughness below 1 nm in both cases (0.53 and 0.40 nm for Figures S4a and S4b, respectively). This indicates that the Silicon surface is almost unaffected by the heat treatments. To favor their comparison, the images have been equalized to the same color scale, from a minimum value of 0 (black) to a maximum of 6 nm (white). In addition to this, Figure S4c shows the Raman spectrum of the substrate in the range 250–350 cm^{-1}, as a function of temperature. Here, we focus on one of the Silicon Raman modes, at 301.65 cm^{-1}, related to the second-order spectrum of transverse (T) acoustical (A) phonons at the critical point X of the Brillouin zone for two-phonon combinations - 2TA(X) [14].

No change in the vibrational mode wavenumber is ever measured at any annealing temperature between 300 and 1000 °C, indicating that there is no stress induced or released by the substrate in the considered heating range.

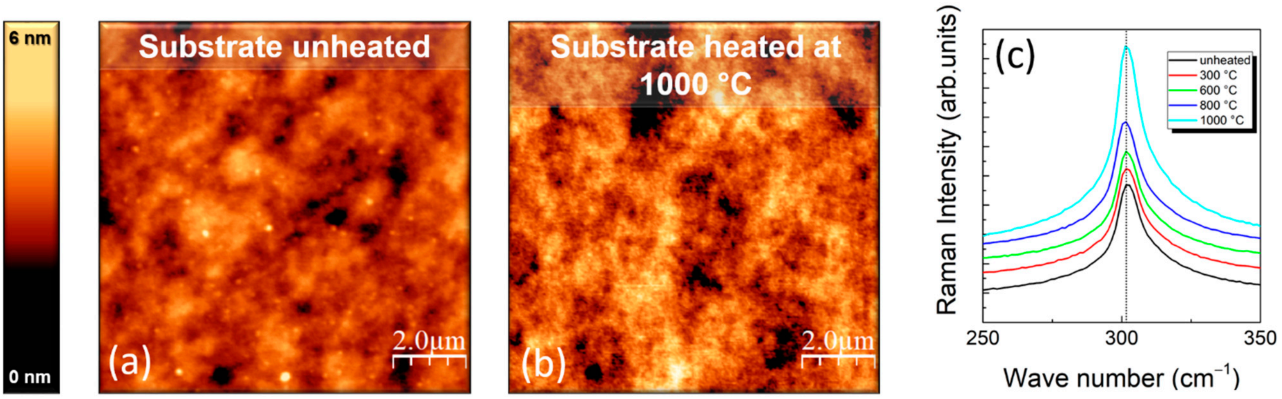

Figure S4. Tapping-mode AFM topography, 10 µm × 10 µm in size, of Silicon substrate (**a**) pristine and (**b**) annealed at 1000 °C. (**c**) Raman spectroscopy of the Silicon substrate unheated (black) and annealed at different temperatures, in the range 250–350 cm^{-1}.

6. Background Correction

Figure S5a shows the untreated (raw data) Raman spectrum of a TiO_2-200 nm film annealed at 800 °C (black curve) and its correspondent Silicon substrate (red curve). The latter has been acquired measuring, each time, the back side of the sample.

The raw data have been treated as follows: first we have removed the contribution of the Silicon substrate by subtracting, point by point, the red curve (Si) from the black curve (TiO_2); then we have normalized the background dividing the obtained TiO_2 Raman spectrum by a linear function, derived from fitting only the tails of the Raman peak (in order to remove any spectrum tilt); finally, we have performed a Lorentzian fit of the as-treated data. In Figure S5b, we plot the normalized Raman spectrum (black scatters) and the corresponding Lorentz fit (red curve), showing a very good agreement between the two. The peak position and the full width at half maximum (FWHM) are extracted from the fit.

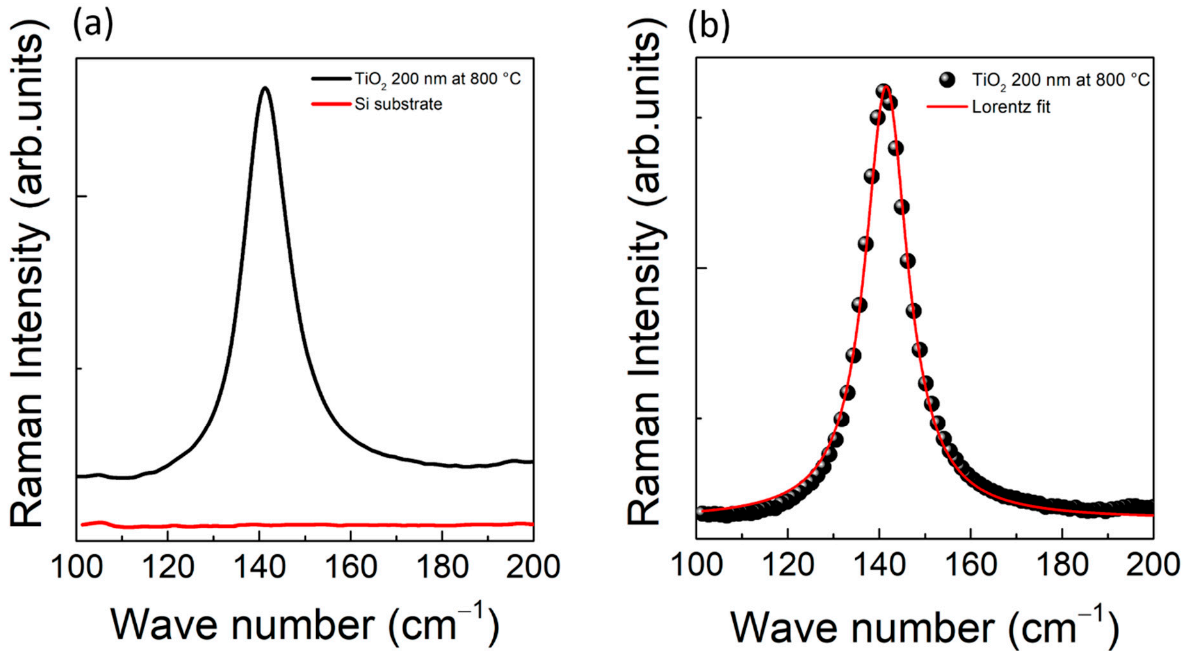

Figure S5. (**a**) Raw Raman data for TiO_2-200 nm film annealed at 800 °C (black curve) and correspondent Silicon substrate (red curve), in the range of interest (100–200 cm^{-1}). (**b**) Raman data of a TiO_2-200 nm film annealed at 800 °C (black scatters) after substrate subtraction and background normalization, and the correspondent Lorentz fit (red curve).

7. Compositional Analysis by EDX

An EDX analysis performed on an as-grown film certifies the absence of any spurious elements. EDX spectra acquired on different regions of a TiO_2-200 nm sample surface are shown in Figure S6. Indeed, only Si (from the substrate), Ti and O have been measured, with the following content in w%: O = 15.10 ± 0.15, Ti = 6.35 ± 0.06, and Si = 78.5 ± 0.1.

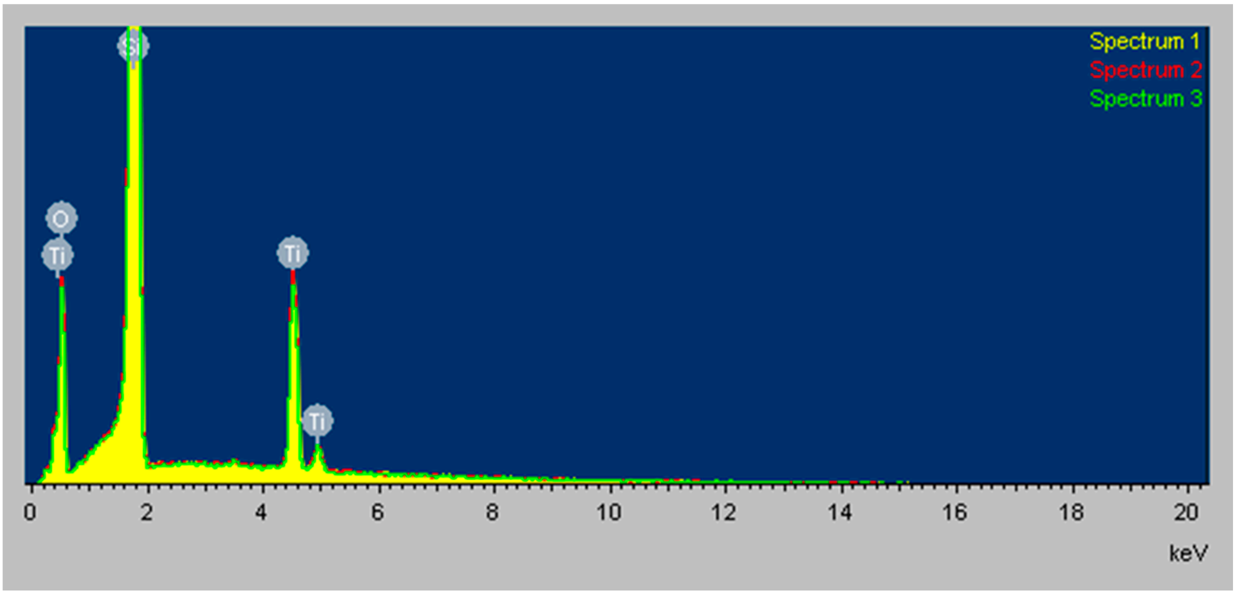

Figure S6. EDX analysis of a TiO_2-200 nm film.

References

1. Albetran, H.; Low, I. Parameters controlling the crystallization kinetics of nanostructured TiO_2 – An overview. Mater. Today: Proc. 2019, 16, 25–35.
2. Shannon, R.D.; Pask, J.A. Kinetics of the Anatase-Rutile Transformation. *J. Am. Ceram. Soc.* **1965**, *48*, 391–398.
3. Eppler, R.A. Effect of Antimony Oxide on the Anatase-Rutile Transformation in Titanium Dioxide. *J. Am. Ceram. Soc.* **1987**, *70*, C-64–C-66.
4. Akhtar, M.K.; Pratsinis, S.E.; Mastrangelo, S.V.R. Dopants in Vapor-Phase Synthesis of Titania Powders. *J. Am. Ceram. Soc.* **1992**, *75*, 3408–341.
5. Ding, X.Z.; Liu, X.H.; He, Y.Z. Grain size dependence of anatase-to-rutile structural transformation in gel-derived nanocrystalline titania powders. *J. Mater. Sci. Lett.* **1996**, *15*, 1789–1791.
6. Gennari, F.C.; Pasquevich, D.M. Kinetics of the anatase–rutile transformation in TiO_2 in the presence of Fe_2O_3. *J. Mater. Sci.* **1998**, *33*, 1571–1578.
7. Zhang, H.; Banfield, J.F. Understanding polymorphic phase transformation behavior during growth of nanocrystalline aggregates: insights from TiO_2. *J. Phys. Chem. B* **2000**, *104*, 3481–3487.
8. Okada, K.; Yamamoto, N.; Kameshima, Y.; Yasumori, A.; MacKenzie, K.J.D. Effect of Silica Additive on the Anatase-to-Rutile Phase Transition. *J. Am. Ceram. Soc.* **2004**, *84*, 1591–1596.
9. Barakat, M.A.; Hayes, G.; Shah, S.I. Effect of Cobalt Doping on the Phase Transformation of TiO_2 Nanoparticles. *J. Nanosci. Nanotechnol.* **2005**, *5*, 759–765.
10. Zhang, H.; Banfield, J.F. Size Dependence of the Kinetic Rate Constant for Phase Transformation in TiO_2 Nanoparticles. *Chem. Mater.* **2005**, *17*, 3421–3425.
11. Fang, D.; Luo, Z.; Huang, K.; Lagoudas, D.C. Effect of heat treatment on morphology, crystalline structure and photocatalysis properties of TiO_2 nanotubes on Ti substrate and freestanding membrane. *Appl. Surf. Sci.* **2011**, *257*, 6451–6461.
12. Liu, R.; Qiang, L.-S.; Yang, W.-D.; Liu, H.-Y. The effect of calcination conditions on the morphology, the architecture and the photo-electrical properties of TiO_2 nanotube arrays. *Mater. Res. Bull.* **2013**, *48*, 1458–1467.
13. Cullity, B.D. *Elements of X-ray Diffraction*, Addison-Wesley Publishing, Boston, MA, USA, 1956.
14. Tanino, H.; Kuprin, A.; Deai, H.; Koshida, N. Raman study of free-standing porous silicon, *Phys. Rev. B* **1996**, *53*, 1937.
